# Supplementary material for: Bayesian modeling reveals metabolite‐dependent ultrasensitivity in the cyanobacterial circadian clock
Source: Mol Syst Biol. 2020 Jun 4;16(6):e9355. doi: 10.15252/msb.20199355 (PMC7271899; doi:10.15252/msb.20199355)
Supplement: Supplementary file 1 — Appendix [file MSB-16-e9355-s001.pdf]

Appendix for

Bayesian Modeling Reveals Metabolite-dependent Ultrasensitivity in the Cyanobacterial Circadian Clock

Lu Hong, Danylo O. Lavrentovich, Archana Chavan, Eugene Leypunskiy, Eileen Li, Charles Matthews, Andy LiWang, Michael J. Rust & Aaron R. Dinner

Contents

1 Additional biochemistry of the KaiC (de)phosphorylation reactions 2

2 The hexameric structure of KaiC 2

3 Correlation structure in the posterior distribution 3

4 Convergence of the model fit 3

5 KaiA function cannot be solely explained in terms of nucleotide exchange 4

6 The experimental data admit two S phosphorylation pathways 4

7 Comparison to the Paijmans model 5

8 Phenomenological modifications to the Phong model 7

List of Figures

Appendix Figure S1 No evidence of direct nucleotide-KaiA interaction

Appendix Figure S2 Performance of the fitting procedure

Appendix Figure S3 Correlation structure in the MCMC ensemble

Appendix Figure S4 The mechanism of kinetic ordering is not well-constrained

Appendix Figure S5 Overview of the model with the multiplicative-factor parametrization scheme

List of Tables

Appendix Table S1 Effects of KaiA on KaiC function

1 Additional biochemistry of the KaiC (de)phosphorylation reactions

In this work we construct a general model of the KaiA-KaiC subsystem based on a set of assumptions of basic clock biochemistry; that is, KaiC is an ATPase and a reversible phosphotransferase with two phosphorylation sites at S431 and T432, while KaiA is a nucleotide-exchange factor that promotes the exchange of bound ADP for ATP in CII nucleotide-binding pockets, and may directly stimulate hydrolysis. Through model fitting, we demonstrate that this set of assumptions is sufficient to explain the (de)phosphorylation kinetics of KaiC and its dependence on %ATP and [KaiA]. Our results, however, do not imply that the model includes all possible biochemical mechanisms; in this section and the next we briefly discuss aspects of KaiC that we do not consider in the model.

First, the current model does not account for the CI domain. The CI domain of KaiC is required for the hexamerization of *S. elongatus* KaiC (Hayashi et al., 2004b, 2006) and its ATPase activity is necessary to allow the binding of KaiB needed to transition into the dephosphorylation phase of the oscillation (Phong et al., 2013; Tseng et al., 2017). However, the ATPase activity of the CI domain has no significant effect on the kinetics of the CII (de)phosphorylation reactions (Phong et al., 2013), and in the current study we are not concerned with KaiB-dependent processes. Therefore the current model does not keep track of the CI hydrolysis state or any allosteric coupling between the CI and CII domains.

Second, the model does not explicitly consider the function of Mg^{2+}. The presence of Mg^{2+} is required for the assembly of the KaiC hexamer (Hayashi et al., 2006; Mutoh et al., 2013), and computational analyses indicate that release of Mg^{2+} independent of the bound nucleotide is highly energetically unfavorable (Hong et al., 2018). Given these results, we assume that Mg^{2+} and nucleotide cannot act independently of each other, and the model implicitly assumes that each bound nucleotide is always in complex with a Mg^{2+} ion. A recent study, however, shows that depletion of Mg^{2+} promotes KaiC autophosphorylation, especially in buffers that lack EDTA, (Jeong et al., 2019). Moreover, some structures of KaiC have two Mg^{2+} ions modeled in the nucleotide-binding pocket, which has been interpreted to mean that KaiC kinase activity relies on a two-metal-ion phosphotransfer mechanism (Pattanayek et al., 2009). Currently, the functions of Mg^{2+} have not been characterized kinetically or mechanistically at a level necessary to constrain its role in a molecularly detailed model.

2 The hexameric structure of KaiC

The current model does not consider any hexameric effects. There is evidence to suggest that intersubunit interaction regulates KaiC autokinase activity (Kitayama et al., 2013) as well as the ultrasensitive dependence of KaiBC complex formation on the KaiC hexamer phosphoform composition (Lin et al., 2014). However, explicitly accounting for the hexameric nature of KaiC, as in Li et al. (2009) or Lin et al. (2014), would lead to a significant increase in the number of model parameters, which likely cannot be constrained by available data and makes interpretation of the model difficult. Therefore, we only keep track of the states of KaiC subunits considered independently, and the rate constants should be considered averages over hexameric background configurations, weighted by their nonequilibrium state populations.

This leads to two simplifications concerning the KaiA-KaiC interactions. The first issue relates to the stoichiometry of KaiAC complexes. During phosphorylation, KaiA dimers bind to KaiC hexamers likely with either a 1:1 or 2:1 stoichiometry (Hayashi et al., 2004a; Yunoki et al., 2019). Because the model does not consider the hexameric structure of KaiC, this stoichiometry is not enforced explicitly, and each KaiC monomer can bind independently to KaiA.

The second issue relates to the effect of the phosphorylation state of the entire hexamer on KaiA (un)binding kinetics. KaiA can inhibit KaiB binding to KaiC (Lin et al., 2014), even though the KaiA and KaiB binding sites are on opposing sides of KaiC. This has been interpreted as a result of cooperative allosteric transition between a kinase mode of KaiC, stabilized by KaiA binding and the T phosphoform, and

a phosphatase mode of KaiC, stabilized by KaiB binding and the S phosphoform (Lin et al., 2014). A possible implication of this proposed mechanism is that KaiC hexamers in the phosphatase mode may have uniformly diminished affinity for KaiA at the CII interface, regardless of the phosphorylation state of the subunits. Given that the current model is trained using primarily the phosphorylation data set, the predicted KaiA dwell time (Figure EV2B) and dissociation constant (Figure 1D) likely reflect the property of KaiC subunits in the kinase mode, whereas experiments with phosphomimetic mutants mimicking the S and D phosphorylation states presumably probe the system kinetics in the phosphatase mode. Indeed, in the KaiC-EE titration experiment (Figure 3F right), the presence of KaiC-EE has virtually no effect on the KaiC S431A stimulus-response curve, which may be partly due to the fact that the KaiC-EE hexamers are in the phosphatase mode and may therefore have additionally reduced affinity for KaiA, a condition not considered in our model.

3 Correlation structure in the posterior distribution

As discussed in the literature (Gutenkunst et al., 2007), often ratios of parameters are better constrained than the parameters themselves. The parameter pairs that have a correlation coefficient larger than 0.9 in log space are shown in Appendix Figure S3A–C. Such correlations typically reflect that thermodynamic, rather than kinetic, properties of the system are constrained. These include the free energy of phosphotransfer between the S and D phosphoforms (Appendix Figure S3A) and the free energy for KaiA binding to the ATP-bound states of KaiC (Appendix Figure S3B; compare with Figure 1D). Interestingly, there is a linear relation among $k_{\mathrm{TP}}^{\mathrm{A,T}}$, $k_{\mathrm{d}}^{\mathrm{A,TU}}$, and $k_{\mathrm{b}}^{\mathrm{T,DP}}$ (Appendix Figure S3C); this implies that the data constrain the flux out of the ${}^{\mathrm{A}}\mathrm{C}_{\mathrm{DP}}^{\mathrm{T}}$ state, but the exact pathway is underdetermined.

More generally, we characterize the extent to which the model parameters, or linear combinations thereof, are constrained by the data using the principal components of the posterior distribution; that is, the eigenvectors of the covariance matrix from MCMC sampling. The eigenvalues of the covariance matrix span multiple orders of magnitude with no obvious gap (Appendix Figure S3D left), except for the stiffest direction (Appendix Figure S3G), which is almost entirely aligned with σ^2, the global error hyperparameter. In addition, the directions of the principal components are in general not aligned with the directions of the bare coordinates (Appendix Figure S3D right), and there is no obvious interpretation for the directions of most of the principal components (Appendix Figure S3G). These features of the ensemble indicate that the model is “sloppy” (Gutenkunst et al., 2007), and many model parameters are poorly constrained by the data. Nevertheless, as we demonstrate in Results, the model can still be used to make consistent predictions because the variabilities in the ensemble of parameter sets obtained from MCMC sampling align with the softest degrees of freedom of the posterior distribution.

4 Convergence of the model fit

We assessed the quality of the fit in three ways. First, we repeated the full procedure three times to assess reproducibility of the fitting procedure (Figure EV1B). The three independent runs yielded marginalized posterior distributions that are remarkably consistent and tightly constrained for some parameters but diverge over several orders of magnitude for others. The best fits from the three runs have log posterior values of 720, 714, and 705, respectively. Unless otherwise specified, in this work we base our analyses on the run that produced the best fit with the highest posterior value. The ruggedness of the posterior distribution demonstrates that given the model and training data set, the parameter estimation problem is far from the asymptotic (i.e., large sample) regime. Moreover, in our fitting procedure the vast majority of the walkers from the annealing step are discarded for the sake of improving the MCMC sampling efficiency (the acceptance rate is $\leq 7\%$ without pruning). Given the presence of multiple local maxima, this choice likely resulted in an underestimation of the uncertainties in parameter values.

Second, we compared the model predictions with a test data set that probed the phosphorylation reac-

Appendix Table S1: Effects of KaiA on KaiC function

Model	Log likelihood			Bayes factor
	Phosphorylation	Dephosphorylation	Hydrolysis	
Full model	422.9	346.8	–0.8	1
–P*	390.0	338.6	–0.8	1.4
–H†	355.5	308.8	–0.9	2.5
–P,–H	278.2	283.6	–0.2	7.7

* –P: KaiA binding decoupled from (de)phosphorylation rates.
† –H: KaiA binding decoupled from KaiC hydrolysis rates.

tion at two non-standard KaiC concentrations (Figure EV2E). The fit quality on the test data set is somewhat worse compared to the training set (compare with Figure 1B). In particular, the model overestimates the D phosphoform concentration at 1.75 μM KaiC and underestimates the T and D phosphoform concentrations at 7 μM KaiC. This result suggests some degree of overfitting. This, however, is not a significant issue because we base our conclusions on the ensemble of walkers rather than the behavior of the best fit.

Lastly, we assessed the convergence of the MCMC simulations using the integrated autocorrelation times for the 48 principal components of the posterior distribution (Appendix Figure S3E). The autocorrelation times for the largest and smallest principal components are 8,500 and 3,300 steps, respectively, which gives rough estimates of the times between independent samples for the slowest and fastest degrees of freedom. These estimates are far shorter than the length of the final MCMC runs in the fitting procedure (100,000 steps). We also checked the autocorrelation time for the KaiA binding affinities (Appendix Figure S3F), which is within the bound given by the principal components.

5 KaiA function cannot be solely explained in terms of nucleotide exchange

Because of the generality of the model, the function of KaiA is not restricted to that of a nucleotide-exchange factor. In particular, the phosphotransfer and ATP hydrolysis rates are allowed to depend on KaiA binding. There is some experimental evidence to support such effects—KaiA binding inhibits dephosphorylation (Xu et al., 2003) and the addition of KaiA increases the ATPase activity of KaiC (Terauchi et al., 2007; Murakami et al., 2008). However, the biochemical mechanisms underlying these effects are not clear from the experiments; for example, does KaiA increase KaiC ATPase activity by reconfiguring the KaiC active site, or does KaiA binding indirectly promote ATP hydrolysis by shifting the KaiC population towards phosphoforms that have high ATPase activity?

To test whether KaiA binding has a direct effect on KaiC catalytic activities, we construct simplified models where hydrolysis and/or phosphotransfer are/is independent of KaiA binding and compare the resulting models to the full model using the Bayes factor (Appendix Table S1). We find that decoupling either phosphotransfer (model –P) or hydrolysis (model –H) from KaiA binding decreases the evidence for the simplified models, but the effects are weak, especially in comparison to a model where both classes of reactions are decoupled from KaiA binding (model –P,–H). These results indicate that the function of KaiA cannot be solely explained by nucleotide exchange, but we cannot conclusively distinguish between models –P and –H.

6 The experimental data admit two S phosphorylation pathways

We analyzed a random selection of 500 walkers to understand the implications of their variations for the mechanisms of ordered phosphorylation. To simplify the analysis, we converted each walker to two single-

site models in which either S431 or T432 was available for phosphorylation but not both. We asked how important each reaction rate constant is to the overall T and S phosphoform concentrations in the single-site models using the relative first-order sensitivities computed at the standard reaction condition (i.e., 100% ATP with 1.5 μM KaiA). We focus on the initial phosphorylation rates because the steady-state rates are determined by balances of many contributing processes, making them harder to interpret. The parameter sensitivities at $t = 1$ h are used as proxies for the sensitivities of the initial phosphorylation rates.

Because each single-site model has 18 parameters, there are 36 sensitivities for the two phosphoforms. To characterize this high-dimensional space, we used spectral clustering (Appendix Figure S4A). Overall, the parameter sensitivities are much more constrained by the data for the T phosphoform than the S phosphoform, which is unsurprising given the relatively low concentrations of the S phosphoform under all experimental conditions in the training data. The clustering furthermore indicates that there are at least two plausible kinetic ordering mechanisms, which differ primarily in terms of the phosphorylation pathways taken by the S phosphoform (Appendix Figure S4 A and B). In both clusters, the U $\rightarrow$ T transition is most sensitive to $k_{\mathrm{p}}^{\mathrm{UT}}$ (i.e., the phosphorylation rate in the absence of KaiA).

In the first cluster (319 parameter sets), the U $\rightarrow$ S transition is most sensitive to $k_{\mathrm{p}}^{\mathrm{A,US}}$ in the presence of KaiA. This is primarily because $k_{\mathrm{p}}^{\mathrm{US}}$ is very small in this cluster relative to $k_{\mathrm{p}}^{\mathrm{A,US}}$ (Appendix Figure S4C); however, since, in this cluster, the U $\rightarrow$ S transition is dominated by the KaiA-bound states, the S phosphoform has negative sensitivity to the KaiA dissociation rate constant $k_{\mathrm{b}}^{\mathrm{U,TP}}$. This suggests that in the first cluster, KaiA (un)binding to the U phosphoform is important in determining the initial phosphorylation rate of S. The best fit belongs to this first cluster.

The phosphorylation pathway suggested by the second cluster (181 parameter sets) is more complex. In this cluster, the U$\rightarrow$S transition is mostly independent of KaiA, similar to the U$\rightarrow$T reaction. However, the S phosphoform is limited by the dephosphorylation reaction $k_{\mathrm{d}}^{\mathrm{SU}}$, which is much faster than the corresponding phosphorylation rate (Appendix Figure S4C). In addition, the S phosphoform is sensitive to the rate constant for KaiA binding, $k_{\mathrm{a}}^{\mathrm{S,DP}}$, which is important for facilitating nucleotide exchange for the ADP-bound S phosphoform, but tends to be slower in the second cluster (Appendix Figure S4C). Therefore, faster dephosphorylation and slower KaiA binding is important for determining the initial S phosphorylation rate in the second cluster.

A comparison of Appendix Figure S4C with Figure EV1B (blue distributions) shows that the two clusters correspond to the bimodal posterior distributions for the rate constants $k_{\mathrm{p}}^{\mathrm{US}}$, $k_{\mathrm{p}}^{\mathrm{A,US}}$, $k_{\mathrm{d}}^{\mathrm{SU}}$, and $k_{\mathrm{a}}^{\mathrm{S,DP}}$. The two clusters, however, do not cleanly separate along the two modes of $k_{\mathrm{a}}^{\mathrm{S,TP}}$ and $k_{\mathrm{b}}^{\mathrm{S,TP}}$; the kinetic significance of this bimodal distribution is unclear.

As discussed above, the posterior distribution is fairly rugged and thus the fitting procedure is not fully reproducible over independent runs. As a result, there are likely multiple potential kinetic ordering mechanisms that remain unexplored through this analysis. Regardless, the analysis suggests that kinetic ordering is likely a result of an interplay between (de)phosphorylation and KaiA (un)binding kinetics, rather than purely the product of equilibrium free energies of phosphotransfer.

7 Comparison to the Paijmans model

Among all the computational work on the Kai oscillator, the model most similar to the current work in terms of the treatment of the KaiA-KaiC subsystem is that by Paijmans et al. (2017), although the latter is a full oscillator model including KaiB, the CI domain, and the allosteric transition between the active (i.e., phopshorylation phase) and inactive (dephosphorylation phase) KaiC conformations. The Paijmans model and the full model in this work are both molecularly detailed, and describe how phosphotransfer, ATP hydrolysis, KaiA (un)binding, and nucleotide exchange reactions control the phosphoform and nucleotide-bound states of KaiC. However, there are some significant differences between these two models, which we

examine below.

The Paijmans model is more general than this work in two ways. First, the Paijmans model explicitly considers the hexameric nature of KaiC. There is no intersubunit coordination of phosphotransfer in the Paijmans model, but it explicitly considers the binding of one KaiA dimer to a KaiC hexamer, which is assumed to uniformly accelerate nucleotide exchange in all six subunits. In this work, however, we do not consider the hexameric states of KaiC, and each KaiC monomer is allowed to bind to a KaiA dimer independently. In this way the affinities and kinetics of KaiA binding in this work may not be directly comparable to those in the Paijmans model. Second, the Paijmans model allows for the exchange of bound ATP for ADP, such that KaiA accelerates the exchange rates of both ATP and ADP while leaving the binding affinity unchanged. In our model, however, we assume that there is no exchange of bound ATP for ADP, effectively assuming that the affinity of KaiC for ATP is infinite (i.e., $K^{\mathrm{CII}}_{\mathrm{ATP/ADP}} = 0$ in the Paijmans model terminology). The treatments of nucleotide exchange in both models are otherwise similar, in that both assume that the ATP/ADP on-rates are identical, that the apo state is in a quasi-steady state, and that there is no KaiA-independent nucleotide exchange.

The current work goes beyond the Paijmans model in the following ways. First, we determine the rate constants under the framework of Bayesian parameter estimation, which enables more rigorous uncertainty quantification, while the parameters in the Paijmans model were hand-tuned to reproduce selected experimental observations. Second, for simplicity the Paijmans model does not consider any possible coupling between ATP hydrolysis and KaiC phosphorylation states, between nucleotide exchange and KaiC phosphorylation states, between ATP hydrolysis and KaiA binding, or between KaiC nucleotide-bound states and KaiA binding. Although many of the state-dependent effects are not fully constrained by data in this work, as we describe in Results, the ultrasensitivity in KaiC phosphorylation depends critically on the coupling between KaiC nucleotide-bound states and KaiA binding. It is an open question whether a model that lacks such effects but explicitly accounts for the hexameric nature of KaiC can generate ultrasensitivity.

The difference in the treatment of KaiA binding affinity implies that some detailed balance conditions are incompatible between the two models. In the Paijmans model, the binding affinity of KaiA to KaiC hexamers during the phosphorylation phase depends on the phosphoform composition of the subunits, and each subunit i in the phosphoform X_i other than U contributes an additive factor of $\delta g^{\mathrm{CII\cdot KaiA}}_{\mathrm{bind}}(\mathrm{X}_i)$ to the changes in KaiA binding free energy, $\Delta G^{\mathrm{CII\cdot KaiA}}_{\mathrm{bind}}$. Due to detailed balance, the fact that KaiA binds to different KaiC phosphoforms with differential affinities implies that KaiA binding changes the free energy of phosphotransfer [see eq. 8 in Paijmans et al. (2017)]. This condition is also present in our model, but is complicated by the nucleotide-bound states of KaiC. Using the multiplicative-factor parametrization scheme (see Materials and Methods), the detailed balance conditions in this work can be related to those in the Paijmans model by

$$\delta g^{\mathrm{CII\cdot KaiA}}_{\mathrm{bind}}(\mathrm{T}) - \delta g^{\mathrm{CII\cdot KaiA}}_{\mathrm{bind}}(\mathrm{U}) = -kT \ln \frac{\delta k^{\mathrm{T,DP}}_{\mathrm{a}}}{\Delta k^{\mathrm{T,DP}}_{\mathrm{b}}} \tag{1}$$

$$\delta g^{\mathrm{CII\cdot KaiA}}_{\mathrm{bind}}(\mathrm{S}) - \delta g^{\mathrm{CII\cdot KaiA}}_{\mathrm{bind}}(\mathrm{U}) = -kT \ln \frac{\delta k^{\mathrm{S,DP}}_{\mathrm{a}}}{\Delta k^{\mathrm{S,DP}}_{\mathrm{b}}} \tag{2}$$

$$\delta g^{\mathrm{CII\cdot KaiA}}_{\mathrm{bind}}(\mathrm{D}) - \delta g^{\mathrm{CII\cdot KaiA}}_{\mathrm{bind}}(\mathrm{T}) = -kT \ln \frac{\Delta k^{\mathrm{D,DP}}_{\mathrm{a}}}{\Delta k^{\mathrm{D,DP}}_{\mathrm{b}}} \frac{\Delta k^{\mathrm{T,TP}}_{\mathrm{b}}}{\delta k^{\mathrm{T,TP}}_{\mathrm{a}}} \tag{3}$$

$$\delta g^{\mathrm{CII\cdot KaiA}}_{\mathrm{bind}}(\mathrm{D}) - \delta g^{\mathrm{CII\cdot KaiA}}_{\mathrm{bind}}(\mathrm{S}) = -kT \ln \frac{\Delta k^{\mathrm{D,DP}}_{\mathrm{a}}}{\Delta k^{\mathrm{D,DP}}_{\mathrm{b}}} \frac{\Delta k^{\mathrm{S,TP}}_{\mathrm{b}}}{\delta k^{\mathrm{S,TP}}_{\mathrm{a}}} \tag{4}$$

In general, this set of equations are inconsistent. That is, one cannot express the $\delta g^{\mathrm{CII\cdot KaiA}}_{\mathrm{bind}}(\mathrm{X})$s in the Paijmans model in terms of the Δks in our model. The only condition under which these equations can be made consistent is when

$$\frac{\delta k^{\mathrm{T,DP}}_{\mathrm{a}}}{\Delta k^{\mathrm{T,DP}}_{\mathrm{b}}} = \frac{\delta k^{\mathrm{T,TP}}_{\mathrm{a}}}{\Delta k^{\mathrm{T,TP}}_{\mathrm{b}}} \quad \text{and} \quad \frac{\delta k^{\mathrm{S,DP}}_{\mathrm{a}}}{\Delta k^{\mathrm{S,DP}}_{\mathrm{b}}} = \frac{\delta k^{\mathrm{S,TP}}_{\mathrm{a}}}{\Delta k^{\mathrm{S,TP}}_{\mathrm{b}}}$$

that is, when the nucleotide-bound states have no effect on KaiA binding affinities to the T and S phospho-

forms.

8 Phenomenological modifications to the Phong model

In the model of Phong et al. (2013), KaiA sequestration is determined by the equation

$$[\mathrm{A}]_{\mathrm{active}} = \max(0, [\mathrm{A}]_{\mathrm{total}} - m[{}^{\mathrm{B}}\mathrm{C}^{\mathrm{S}}] - n[{}^{\mathrm{B}}\mathrm{C}^{\mathrm{D}}]). \tag{5}$$

Here, $[\mathrm{A}]_{\mathrm{total}}$ is the total KaiA concentration; $[{}^{\mathrm{B}}\mathrm{C}^{\mathrm{S}}]$ and $[{}^{\mathrm{B}}\mathrm{C}^{\mathrm{D}}]$ are the concentrations of the S and D phosphoforms in complex with KaiB, respectively; m and n are model parameters describing the binding stoichiometries between KaiA and the KaiBC complex. In this way, the KaiA binding affinity to the inhibitory complex is effectively infinite, with no KaiA dissociation until $[{}^{\mathrm{B}}\mathrm{C}^{\mathrm{S}}]$ and $[{}^{\mathrm{B}}\mathrm{C}^{\mathrm{D}}]$ drop below a threshold. To make the representation of KaiA sequestration more realistic, we introduce a KaiA dissociation constant K_D (Figure 4C). Similar to the treatment in Kim and Forger (2012), we assume that the KaiA sequestration reaction is in a quasi-equilibrium and replace (5) with

$$[\mathrm{A}]_{\mathrm{active}} = \frac{1}{2}\left([\mathrm{A}]_{\mathrm{total}} - \mathrm{SD} - K_D + \sqrt{4[\mathrm{A}]_{\mathrm{total}}K_D + ([\mathrm{A}]_{\mathrm{total}} - \mathrm{SD} - K_D)^2}\right) \tag{6}$$

where we have defined $\mathrm{SD} = m[{}^{\mathrm{B}}\mathrm{C}^{\mathrm{S}}] + n[{}^{\mathrm{B}}\mathrm{C}^{\mathrm{D}}]$.

To introduce ultrasensitivity to the Phong model, we first note that the four phosphorylation rate constants for the U→T, U→S, T→D, and S→D transitions are given by Michaelis-Menten kinetics with ADP serving as a competitive inhibitor,

$$k_{\mathrm{phos}} = \frac{k^{\mathrm{A}}_{\mathrm{phos}}[\mathrm{A}]_{\mathrm{active}}}{K_{1/2} + [\mathrm{A}]_{\mathrm{active}}}\frac{1}{1 + K_I[\mathrm{ADP}]/[\mathrm{ATP}]} \tag{7}$$

where $k^{\mathrm{A}}_{\mathrm{phos}}$ varies with the specific phosphorylation reaction. To introduce ultrasensitivity, we add a threshold term T (Figure 4D),

$$k_{\mathrm{phos}} = \frac{k^{\mathrm{A}}_{\mathrm{phos}}([\mathrm{A}]_{\mathrm{active}} - T)}{K_{1/2} + ([\mathrm{A}]_{\mathrm{active}} - T)}\frac{1}{1 + K_I[\mathrm{ADP}]/[\mathrm{ATP}]}H([\mathrm{A}]_{\mathrm{active}} - T) \tag{8}$$

where

$$T = (0.3 + 1.5e^{-5\%\mathrm{ATP}/100\%})(1 + 0.08[\mathrm{C}^{\mathrm{U}}]/\mu\mathrm{M}) \tag{9}$$

and H is the Heaviside function. The first part of the expression for the phosphorylation threshold, $0.3 + 1.5e^{-5\%\mathrm{ATP}/100\%}$, describes how the threshold changes as a function of %ATP; the constants are determined by approximating the [KaiA] threshold in Figure 3A as an exponential function. The second part of the expression, $1 + 0.08[\mathrm{C}^{\mathrm{U}}]/\mu\mathrm{M}$, describes how the threshold changes as a function of C^{U} concentration. This formula is defined by

$$\frac{a/\mu\mathrm{M} + b([\mathrm{C}^{\mathrm{U}}]/\mu\mathrm{M} - 3.5)}{a/\mu\mathrm{M} + b(-3.5)}$$

where parameters a and b are determined by taking a linear fit, $a + b[\mathrm{AA}]$, of the data from Figure 3G (yellow line); that is, $1 + 0.08[\mathrm{C}^{\mathrm{U}}]/\mu\mathrm{M}$ gives the fractional changes to the phosphorylation threshold as a result of any U phosphoform (in the form of AA phosphomimetic mutant) additional to the 3.5 μM KaiC S431A in the experiment.

We use the original Phong model parameters in all analyses of the model with one exception. In the final modified model with both a K_D and a phosphorylation threshold (Figure 4E bottom), the period is systematically longer than 24 h due to a slow down in phosphorylation. To fix this problem, we change k^{A}_{ds} and k^{0}_{ds}, the two rate constants controlling the D→S transition, to 0.94 k^{A}_{ds} and 1.1 k^{0}_{ds}.

To assess whether the ultrasensitivity we introduced into the model with (8) is consistent with our measurements, we simulated the KaiC-AA titration experiment using the modified Phong model (Figure EV3E) and compared the predicted EC50 with the experimental results (Figure 3G). To perform this simulation, we set all phosphorylation rates to the S and D states to zero, and represent KaiC-AA concentration as a constant offset to the $[C^U]$ term in (9). A comparison of the model prediction with the experimental result shows that EC50 scales with [AA] in the model similarly to the experiment, but the EC50 in the model is consistently larger than the experiment by about 0.15 µM. This result suggests that the ultrasensitivity we introduced into the model by hand is reasonable, but to achieve precise agreement with experiment would require fine tuning the Phong model parameters, which is outside the scope of the current work.

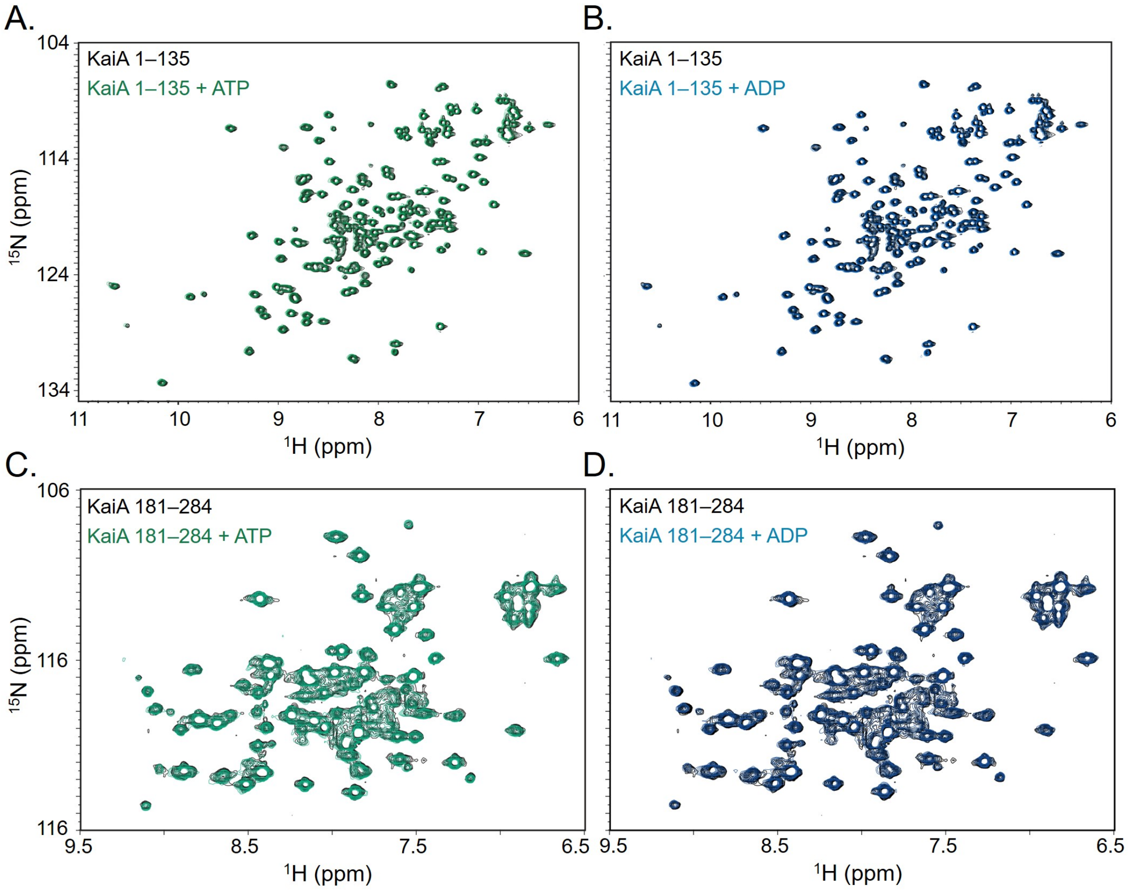

Appendix Figure S1: No evidence of direct nucleotide-KaiA interaction. ^{1}H-^{15}N HSQC spectra of the N-terminal fragment (residues 1–135) of KaiA in the presence and absence of ATP (A) or ADP (B) show no significant differences in chemical shifts, while spectra of the C-terminal fragment (residues 181–284) show subtle line broadening in the presence of ATP (C) and ADP (D), suggesting weak, if any, interaction between the nucleotide and the C-terminal fragment. Given these results, we do not include any direct KaiA-nucleotide interaction in the model.

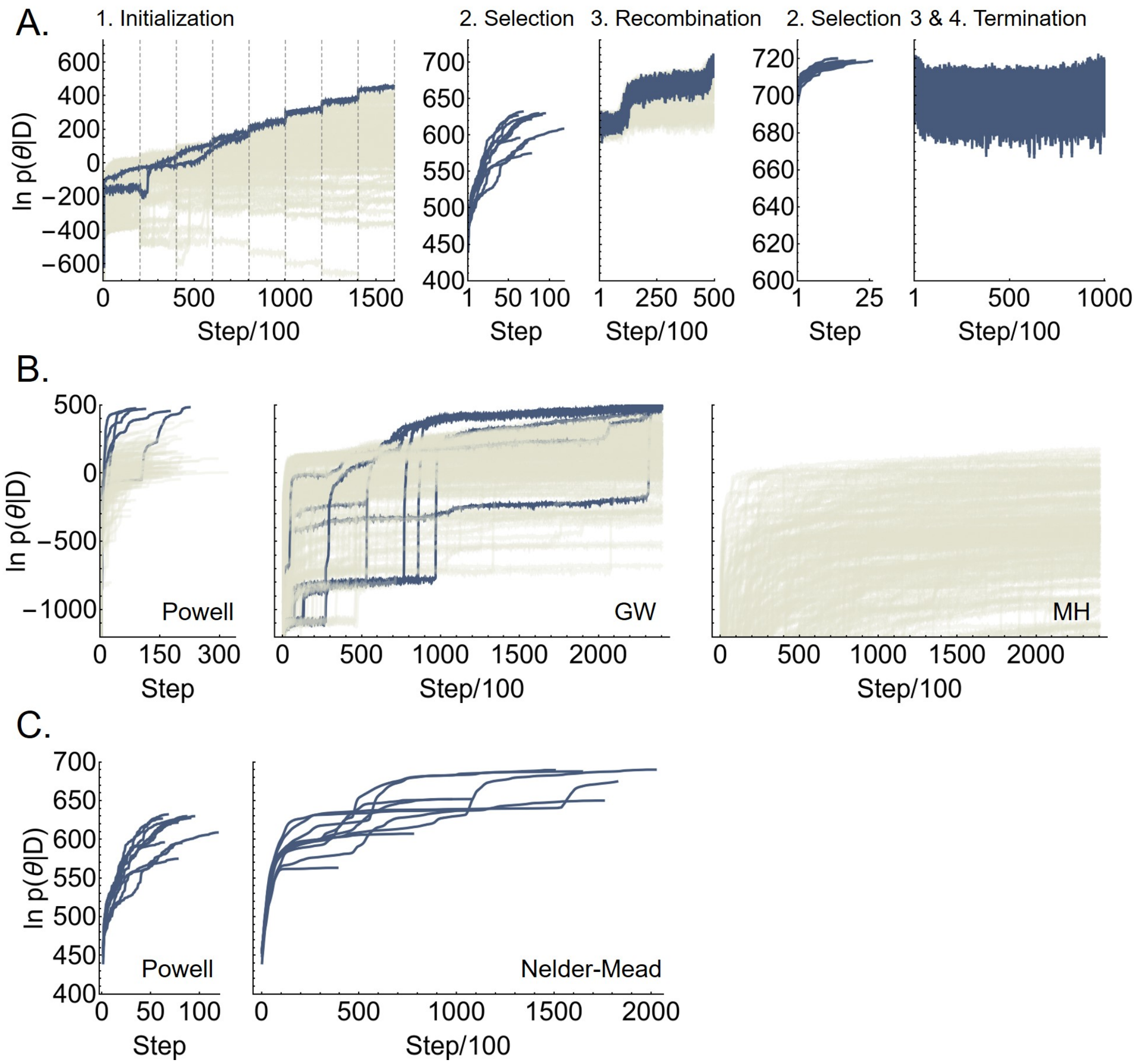

Appendix Figure S2: Performance of the fitting procedure. A) The time evolution of the log posterior values over the four steps of the fitting procedure (see Materials and Methods). For step 1 and 3, the individual Markov chains that do not produce walkers used in the next step are shown in beige. B) A comparison of the performance of Powell's method, a derivative-free numerical optimization method, Goodman-Weare (GW) ensemble MCMC method, and conventional Metropolis-Hastings (MH) algorithm with a Gaussian trial distribution. For the Metropolis-Hastings algorithm the covariance matrix of the trial distribution is given by the global covariance of the fit (i.e., the last step in panel A), scaled by a factor of 0.005 to give an average acceptance rate of 19.8%. A set of 224 walkers drawn from the prior distribution are used to initialize the simulations for all three methods; the 224 walkers are evolved independently for the Powell's and Metropolis-Hastings methods, and in an ensemble for the Goodman-Weare method. Chains that do not reach log posterior above 450 are shown in beige. C) A comparison of the performance of Powell's method with the Nelder-Mead simplex-based numerical optimization method. The simulations are initialized using the same walkers as in step 2 of A).

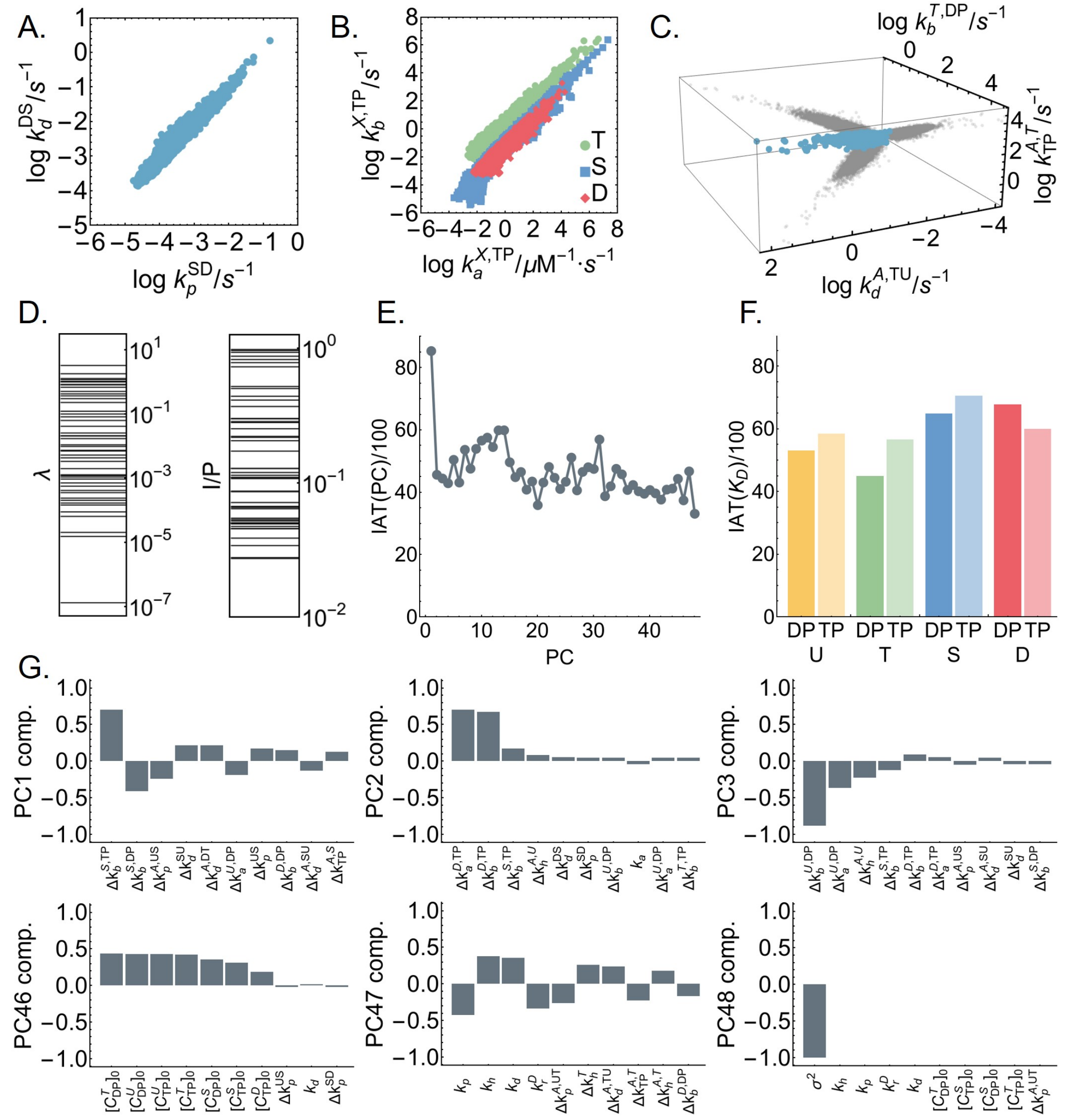

Appendix Figure S3: Correlation structure in the MCMC ensemble. A), B), and C) show parameters with correlation coefficients larger than 0.9. In B), "X" represents the KaiC phosphoforms. In C), the projections of the 3D scatter plot onto pairwise correlations are shown in gray. D) The principal component/eigenvalue spectrum of the covariance matrix (left), and the alignment of the principal components with the coordinates (right). Here, I denotes the intersection of the principal component ellipoid with the coordinates and P denotes the projection of the principal components onto the corresponding coordinates (Gutenkunst et al., 2007). E) The integrated autocorrelation time for the 48 principal components (PC); the principal components are indexed from the largest to the smallest. The integrated autocorrelation time is calculated using an automated windowing procedure (Madras and Sokal, 1988) from the autocorrelation function averaged over the ensemble. F) The integrated autocorrelation time for the KaiA dissociation constants as a function of KaiC phosphoform and nucleotide-bound states. G) The ten largest vector components, ordered by absolute value, for the first and last three principal components.

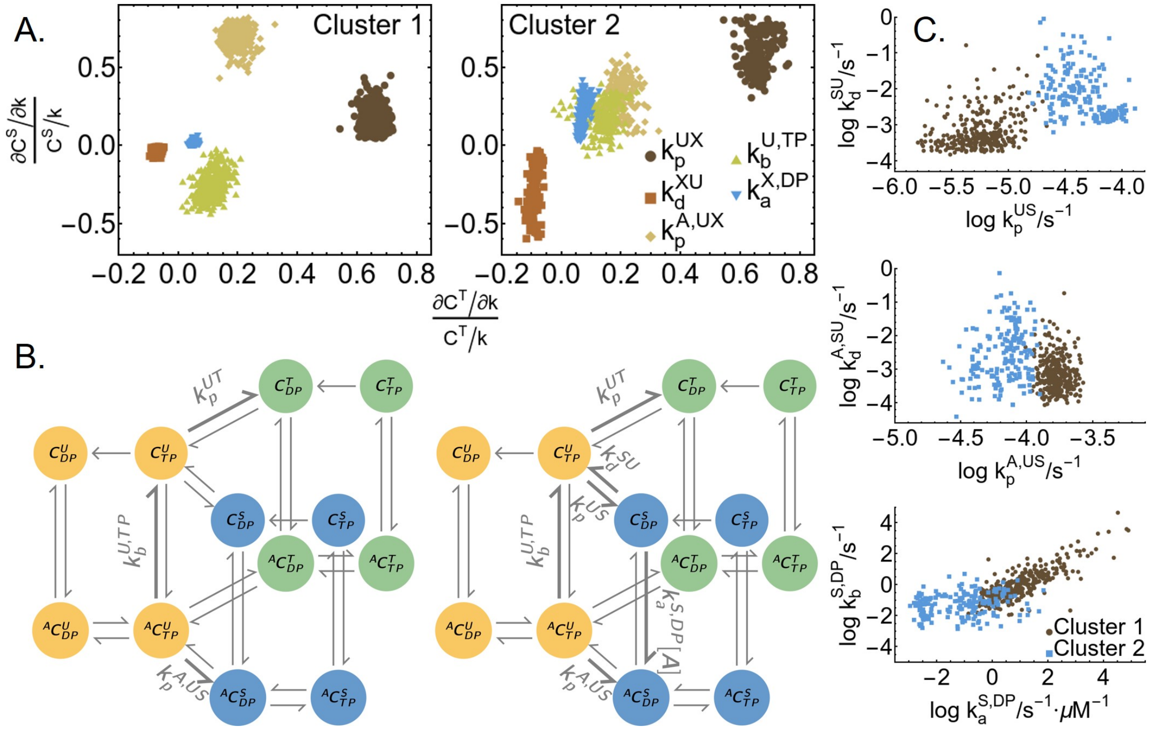

Appendix Figure S4: The mechanism of kinetic ordering is not well-constrained. A) Spectral clustering on the relative sensitivity of the T and S phosphoform concentrations at $t = 1$ h to rate constants in the T- and S-site models, respectively. Only the parameters with significant (> 0.2) relative sensitivities in either cluster are shown in the plot. "X" stands for either the T (horizontal axis) or S (vertical axis) phosphoform. The sensitivities are calculated using 500 sampled parameter sets chosen randomly from the ensemble. The clustering analysis was done using the FindClusters function in Mathematica 12.0. B) Model diagrams that highlight the reactions that have the highest relative sensitivities in the first (left) and second (right) clusters; the D phosphoform is not shown for clarity. C) Selected model parameter values in the two clusters. A comparison with blue distributions in Figure EV1B indicates that the clustering based on sensitivity can be mapped onto the modes of the posterior distribution.

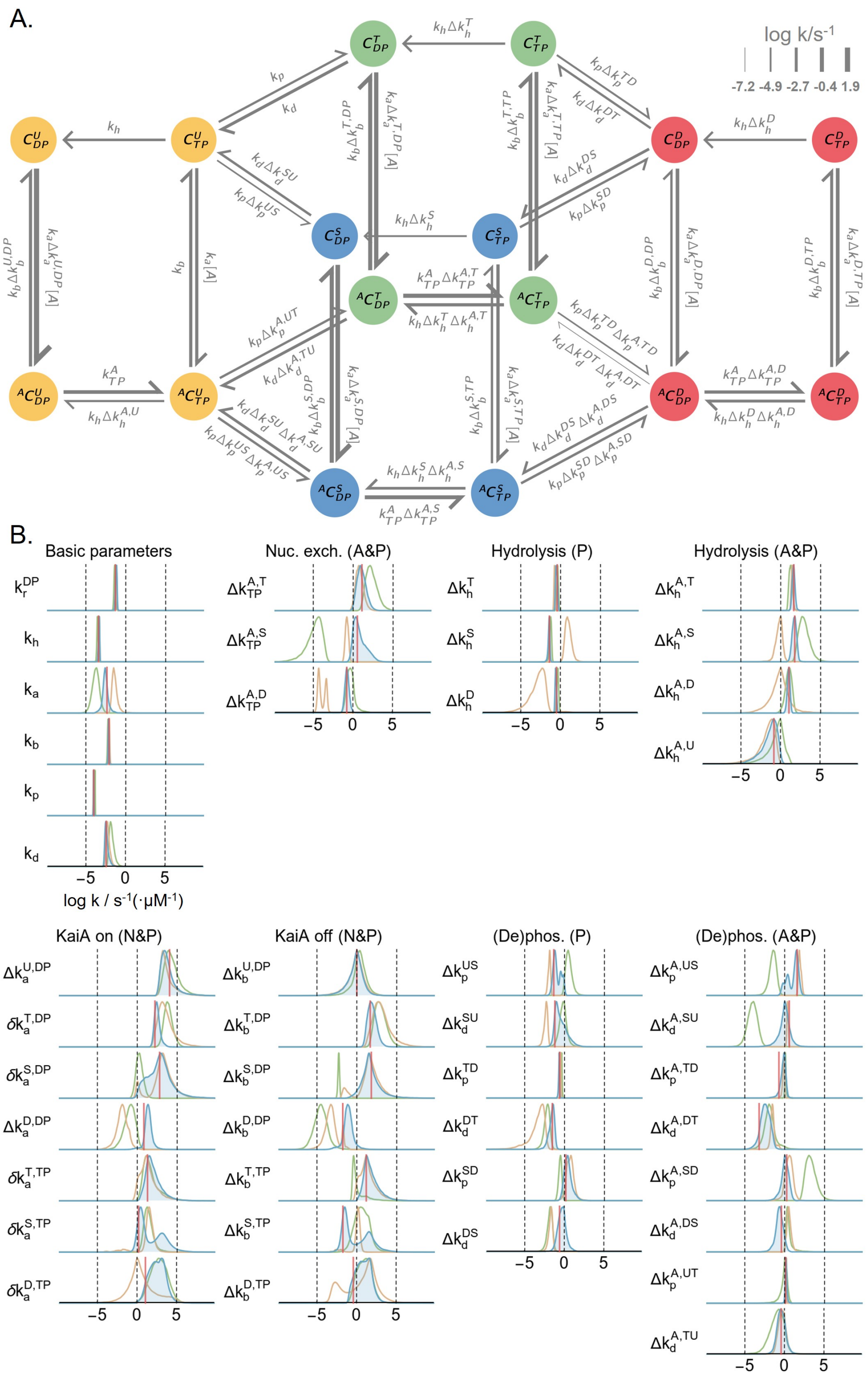

Appendix Figure S5: Overview of the model with the multiplicative-factor parameterization scheme. Panels A) and B) are analogous to those in Figure EV1, but the rate constants are represented as products of the factors that are actually optimized in the MCMC simulations. In B), the δk parameters are fixed parameters determined by detailed balance conditions. The parentheses denote state-dependent effects; A: KaiA-bound state, P: phosphoform, N: nucleotide-bound state. The horizontal axis has a log scale (base 10). The rate constants have the unit s^{-1} (or $s^{-1}\cdot\mu M^{-1}$ for the KaiA on rate constant k_a) and the multiplicative factors are dimensionless. See Materials and Methods for further description of the detailed balance conditions and the model parameterization method.

References

Gutenkunst, R. N., Waterfall, J. J., Casey, F. P., Brown, K. S., Myers, C. R. and Sethna, J. P. (2007) Universally Sloppy Parameter Sensitivities in Systems Biology Models. *PLOS Computational Biology*, **3**, e189.

Hayashi, F., Ito, H., Fujita, M., Iwase, R., Uzumaki, T. and Ishiura, M. (2004a) Stoichiometric interactions between cyanobacterial clock proteins KaiA and KaiC. *Biochemical and Biophysical Research Communications*, **316**, 195–202.

Hayashi, F., Itoh, N., Uzumaki, T., Iwase, R., Tsuchiya, Y., Yamakawa, H., Morishita, M., Onai, K., Itoh, S. and Ishiura, M. (2004b) Roles of Two ATPase-Motif-containing Domains in Cyanobacterial Circadian Clock Protein KaiC. *Journal of Biological Chemistry*, **279**, 52331–52337.

Hayashi, F., Iwase, R., Uzumaki, T. and Ishiura, M. (2006) Hexamerization by the N-terminal domain and intersubunit phosphorylation by the C-terminal domain of cyanobacterial circadian clock protein KaiC. *Biochemical and Biophysical Research Communications*, **348**, 864–872.

Hong, L., Vani, B. P., Thiede, E. H., Rust, M. J. and Dinner, A. R. (2018) Molecular dynamics simulations of nucleotide release from the circadian clock protein KaiC reveal atomic-resolution functional insights. *Proceedings of the National Academy of Sciences*, 201812555.

Jeong, Y. M., Dias, C., Diekman, C., Brochon, H., Kim, P., Kaur, M., Kim, Y.-S., Jang, H.-I. and Kim, Y.-I. (2019) Magnesium Regulates the Circadian Oscillator in Cyanobacteria. *Journal of Biological Rhythms*, 0748730419851655.

Kim, J. K. and Forger, D. B. (2012) A mechanism for robust circadian timekeeping via stoichiometric balance. *Molecular Systems Biology*, **8**, 630.

Kitayama, Y., Nishiwaki-Ohkawa, T., Sugisawa, Y. and Kondo, T. (2013) KaiC intersubunit communication facilitates robustness of circadian rhythms in cyanobacteria. *Nature Communications*, **4**, 2897.

Li, C., Chen, X., Wang, P. and Wang, W. (2009) Circadian KaiC Phosphorylation: A Multi-Layer Network. *PLOS Comput Biol*, **5**, e1000568.

Lin, J., Chew, J., Chockanathan, U. and Rust, M. J. (2014) Mixtures of opposing phosphorylations within hexamers precisely time feedback in the cyanobacterial circadian clock. *Proceedings of the National Academy of Sciences*, **111**, E3937–E3945.

Madras, N. and Sokal, A. D. (1988) The pivot algorithm: A highly efficient Monte Carlo method for the self-avoiding walk. *Journal of Statistical Physics*, **50**, 109–186.

Murakami, R., Miyake, A., Iwase, R., Hayashi, F., Uzumaki, T. and Ishiura, M. (2008) ATPase activity and its temperature compensation of the cyanobacterial clock protein KaiC. *Genes to Cells*, **13**, 387–395.

Mutoh, R., Nishimura, A., Yasui, S., Onai, K. and Ishiura, M. (2013) The ATP-Mediated Regulation of KaiB-KaiC Interaction in the Cyanobacterial Circadian Clock. *PLOS ONE*, **8**, e80200.

Paijmans, J., Lubensky, D. K. and ten Wolde, P. R. (2017) A thermodynamically consistent model of the post-translational Kai circadian clock. *PLOS Computational Biology*, **13**, e1005415.

Pattanayek, R., Mori, T., Xu, Y., Pattanayek, S., Johnson, C. H. and Egli, M. (2009) Structures of KaiC Circadian Clock Mutant Proteins: A New Phosphorylation Site at T426 and Mechanisms of Kinase, ATPase and Phosphatase. *PLOS ONE*, **4**, e7529.

Phong, C., Markson, J. S., Wilhoite, C. M. and Rust, M. J. (2013) Robust and tunable circadian rhythms from differentially sensitive catalytic domains. *Proceedings of the National Academy of Sciences*, **110**, 1124–1129.

Terauchi, K., Kitayama, Y., Nishiwaki, T., Miwa, K., Murayama, Y., Oyama, T. and Kondo, T. (2007) ATPase activity of KaiC determines the basic timing for circadian clock of cyanobacteria. *Proceedings of the National Academy of Sciences*, **104**, 16377–16381.

Tseng, R., Goularte, N. F., Chavan, A., Luu, J., Cohen, S. E., Chang, Y.-G., Heisler, J., Li, S., Michael, A. K., Tripathi, S., Golden, S. S., LiWang, A. and Partch, C. L. (2017) Structural basis of the day-night transition in a bacterial circadian clock. *Science*, **355**, 1174–1180.

Xu, Y., Mori, T. and Johnson, C. H. (2003) Cyanobacterial circadian clockwork: Roles of KaiA, KaiB and the kaiBC promoter in regulating KaiC. *The EMBO Journal*, **22**, 2117–2126.

Yunoki, Y., Ishii, K., Yagi-Utsumi, M., Murakami, R., Uchiyama, S., Yagi, H. and Kato, K. (2019) ATP hydrolysis by KaiC promotes its KaiA binding in the cyanobacterial circadian clock system. *Life Science Alliance*, **2**, e201900368.

All parameter values are given in log space (base 10). All first-order rate constants have units of s^{-1}, and all second-order rate constants have units of $s^{-1} \cdot \mu M^{-1}$. The multiplicative factors (Δk) are dimensionless.

Table S2: Best fit parameter values (independent-rate scheme)

k_h^{U}	−3.409
k_h^{T}	−3.837
k_h^{S}	−4.816
k_h^{D}	−3.895
$k_h^{A,U}$	−4.242
$k_h^{A,T}$	−2.203
$k_h^{A,S}$	−3.066
$k_h^{A,D}$	−2.869
$k_r^{DP,U}$	−1.294
$k_r^{DP,T}$	−0.1942
$k_r^{DP,S}$	−0.7744
$k_r^{DP,D}$	−2.072
k_p^{UT}	−4.005
k_d^{TU}	−2.434
k_p^{US}	−5.360
k_d^{SU}	−3.650
k_p^{TD}	−4.629
k_d^{DT}	−3.969
k_p^{SD}	−3.842
k_d^{DS}	−3.033

$k_p^{A,UT}$	−3.881
$k_d^{A,TU}$	−2.850
$k_p^{A,US}$	−3.804
$k_d^{A,SU}$	−3.082
$k_p^{A,TD}$	−5.352
$k_d^{A,DT}$	−7.174
$k_p^{A,SD}$	−3.587
$k_d^{A,DS}$	−3.449
$k_a^{U,TP}$	−2.352
$k_a^{U,DP}$	1.697
$k_a^{T,TP}$	−1.068
$k_a^{T,DP}$	−0.1370
$k_a^{S,TP}$	−2.183
$k_a^{S,DP}$	0.4483
$k_a^{D,TP}$	−1.288
$k_a^{D,DP}$	−1.528
$k_b^{U,TP}$	−2.093
$k_b^{U,DP}$	−2.093
$k_b^{T,TP}$	−0.9147
$k_b^{T,DP}$	−0.4178
$k_b^{S,TP}$	−3.840
$k_b^{S,DP}$	−0.2805
$k_b^{D,TP}$	−2.504
$k_b^{D,DP}$	−3.856

Table S3: Best fit parameter values (multiplicative-factor scheme)

k_r^{DP}	-1.294
k_h	-3.409
k_a	-2.352
k_b	-2.093
k_p	-4.005
k_d	-2.434
$\Delta k_{\mathrm{TP}}^{\mathrm{A,T}}$	1.100
$\Delta k_{\mathrm{TP}}^{\mathrm{A,S}}$	0.5193
$\Delta k_{\mathrm{TP}}^{\mathrm{A,D}}$	-0.7781
Δk_h^{T}	-0.4280
Δk_h^{S}	-1.407
Δk_h^{D}	-0.4861
$\Delta k_h^{\mathrm{A,T}}$	1.634
$\Delta k_h^{\mathrm{A,S}}$	1.750
$\Delta k_h^{\mathrm{A,D}}$	1.026
$\Delta k_h^{\mathrm{A,U}}$	-0.8333

$\Delta k_a^{\mathrm{U,DP}}$	4.049
$\delta k_a^{\mathrm{T,DP}}$	2.215
$\delta k_a^{\mathrm{S,DP}}$	2.801
$\Delta k_a^{\mathrm{D,DP}}$	0.8245
$\delta k_a^{\mathrm{T,TP}}$	1.284
$\delta k_a^{\mathrm{S,TP}}$	0.1691
$\delta k_a^{\mathrm{D,TP}}$	1.064
$\Delta k_b^{\mathrm{U,DP}}$	0.000 124 7
$\Delta k_b^{\mathrm{T,DP}}$	1.676
$\Delta k_b^{\mathrm{S,DP}}$	1.813
$\Delta k_b^{\mathrm{D,DP}}$	-1.763
$\Delta k_b^{\mathrm{T,TP}}$	1.179
$\Delta k_b^{\mathrm{S,TP}}$	-1.746
$\Delta k_b^{\mathrm{D,TP}}$	-0.4111
Δk_p^{US}	-1.355
Δk_d^{SU}	-1.216
Δk_p^{TD}	-0.6240
Δk_d^{DT}	-1.535
Δk_p^{SD}	0.1626
Δk_d^{DS}	-0.5983
$\Delta k_p^{\mathrm{A,US}}$	1.556
$\Delta k_d^{\mathrm{A,SU}}$	0.5683
$\Delta k_p^{\mathrm{A,TD}}$	-0.7233
$\Delta k_d^{\mathrm{A,DT}}$	-3.205
$\Delta k_p^{\mathrm{A,SD}}$	0.2554
$\Delta k_d^{\mathrm{A,DS}}$	-0.4163
$\Delta k_p^{\mathrm{A,UT}}$	0.1239
$\Delta k_d^{\mathrm{A,TU}}$	-0.4157
