# Supplementary material for: Bayesian modeling reveals metabolite‐dependent ultrasensitivity in the cyanobacterial circadian clock
Source: Mol Syst Biol. 2020 Jun 4;16(6):e9355. doi: 10.15252/msb.20199355 (PMC7271899; doi:10.15252/msb.20199355)
Supplement: Supplementary file 2 — Expanded View Figures PDF [file MSB-16-e9355-s002.pdf]

Expanded View Figures

Figure EV1. Overview of the model.

A A schematic of the full mass-action kinetic model. Here, each arrow represents a reaction, and the associated rate constant is represented using the notation introduced in the main text. The thickness of the arrows is proportional to the best fit rate on a log scale (base 10) at 100% ATP and 1.5 μM KaiA.

B The posterior distributions for all rate constants, initial conditions, and the global error hyperparameter. The three distributions represent the results from three independent runs; the log-posterior values for the best fits from the three runs are listed. The red lines represent the best fit from the best run (i.e., the blue distributions). See Materials and Methods and Appendix Fig S5 for further details on the model parameterization method.

 © 2020 The Authors

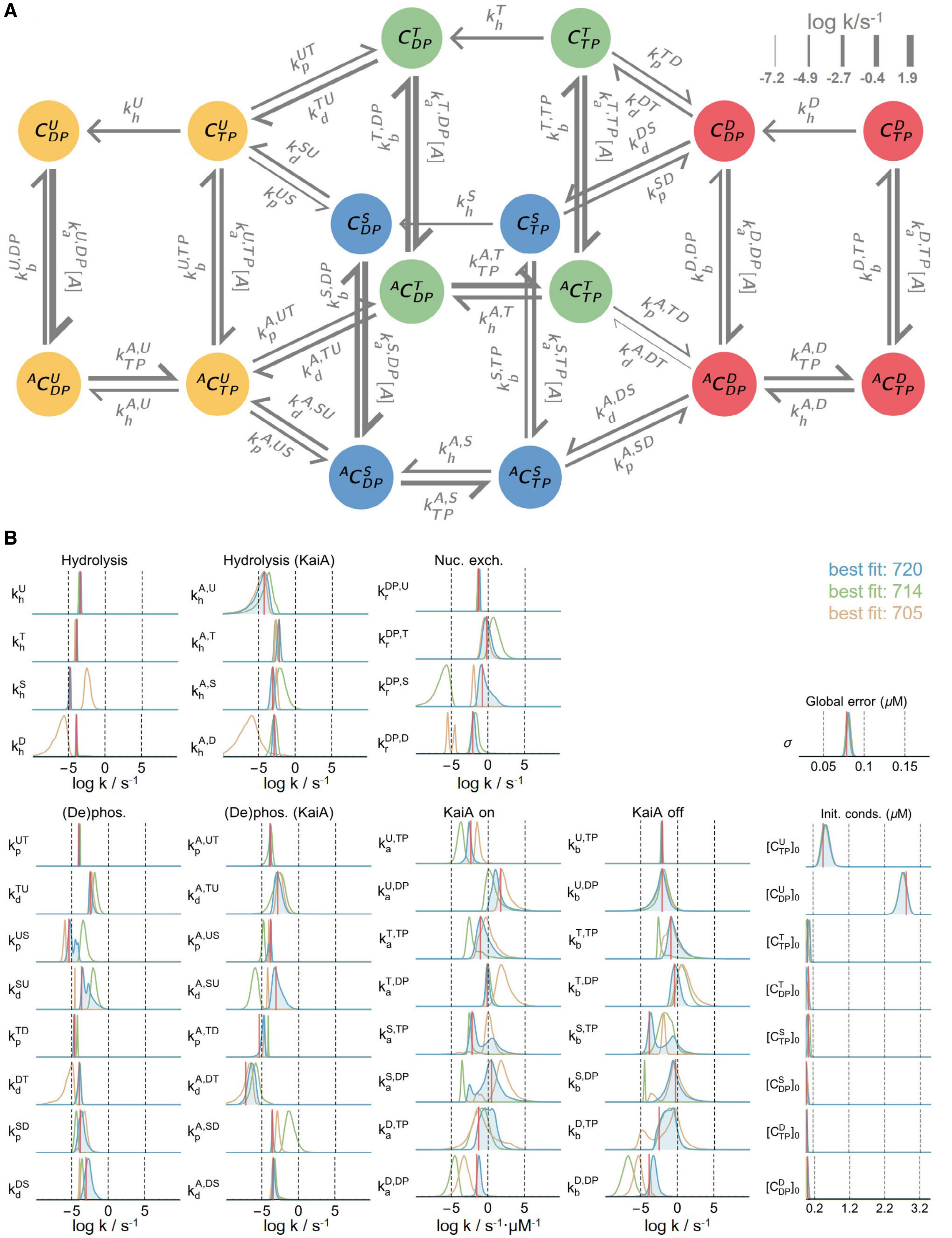
A
log k/s-1
-7.2 -4.9 -2.7 -0.4 1.9
B
Hydrolysis
Hydrolysis (KaiA)
Nuc. exch.
best fit: 720
best fit: 714
best fit: 705
Global error (μM)
σ
0.05 0.1 0.15
log k / s-1
(De)phos.
(De)phos. (KaiA)
KaiA on
KaiA off
Init. conds. (μM)
log k / s-1·μM-1
0.2 1.2 2.2 3.2

Figure EV1.

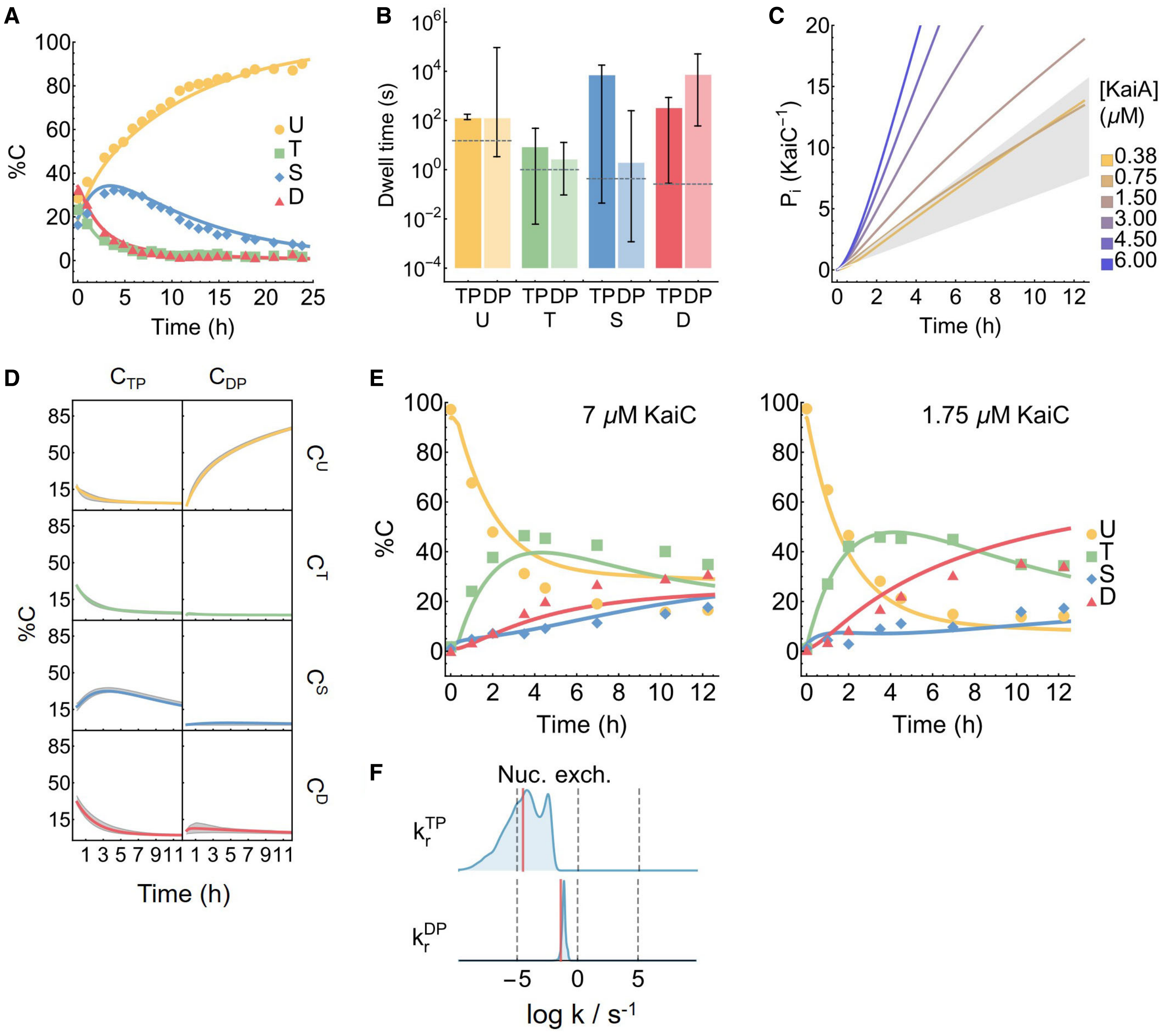

Figure EV2. Behavior of the model.

A Model fit to the dephosphorylation dataset (Rust *et al*, 2007).

B The best fit KaiA dwell time as a function of KaiC phosphoform and nucleotide-bound state. The error bars represent the 95% posterior interval, computed from the final 30,000 MCMC steps of the 224 walkers of the ensemble of the inference run, shown in blue in Fig EV1B. The dashed lines represent the experimental measurements (Kageyama *et al*, 2006; Mori *et al*, 2018), which did not resolve the nucleotide-bound states.

C Inorganic phosphate production per KaiC monomer over the course of a phosphorylation reaction. The gray region represents the experimental bounds on the KaiC hydrolysis rate with 1.2 μM KaiA and no KaiA (Terauchi *et al*, 2007).

D The kinetics of the dephosphorylation reaction in the absence of KaiA, broken down into the eight individual KaiC states. The gray region represents the 95% posterior interval. Refer to Fig EV1A for the KaiC state names.

E The predicted phosphorylation kinetics at 7 and 1.75 μM KaiC, both at 100% ATP and 1.5 μM KaiA, compared to experimental measurements. Note that these two time series are not part of the training set.

F The posterior distributions for k_r^{TP} and k_r^{DP}, the dissociation rates for ATP and ADP, respectively, in an early iteration of the model. The horizontal axis is on a base 10 log scale. The long tail to the left of the posterior distribution for k_r^{TP} suggests that the model can be simplified by setting the rate to zero.

 © 2020 The Authors

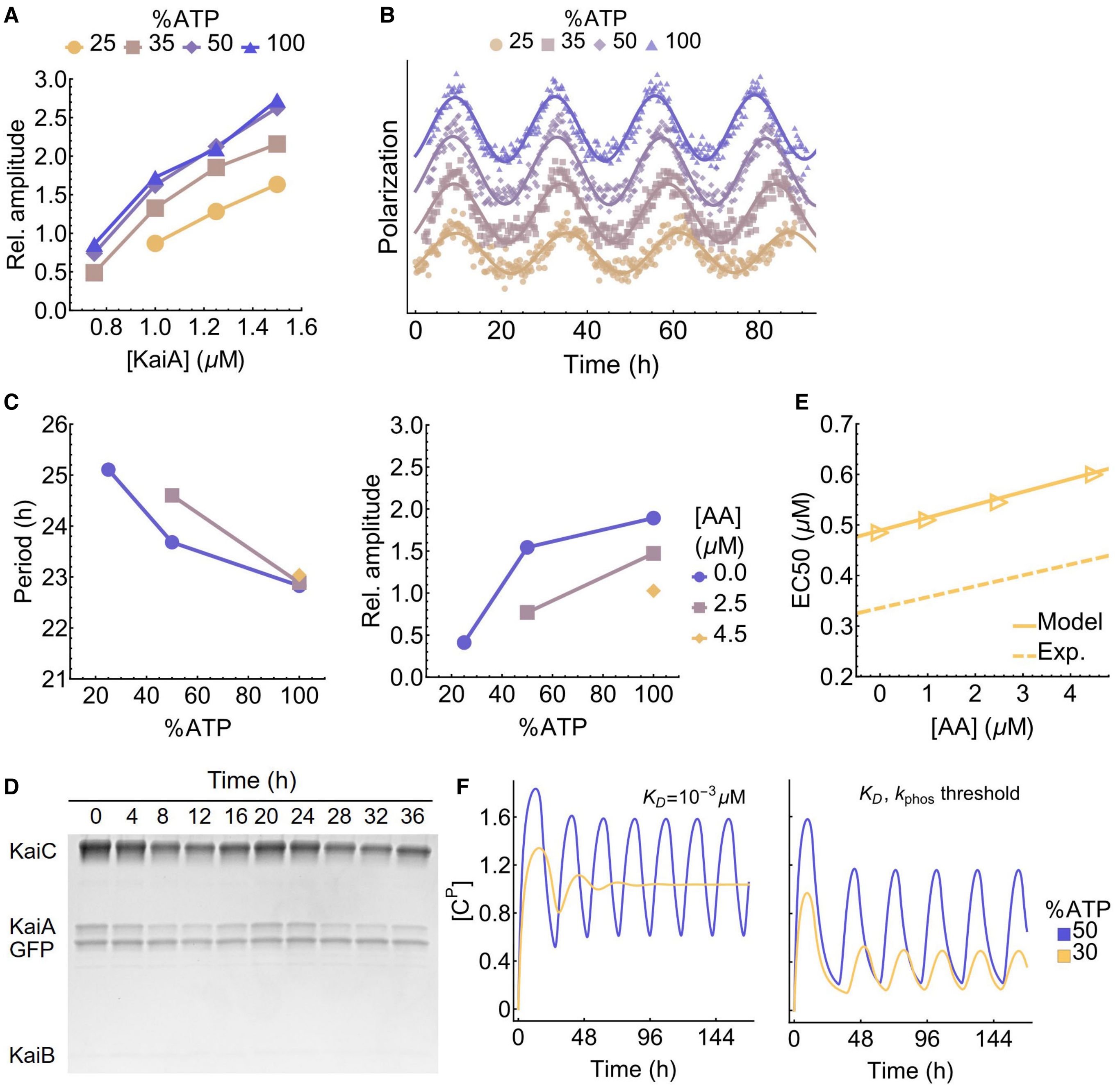

Figure EV3. The metabolic compensation property of the Kai oscillator.

A Fluorescence polarization measurements of oscillatory reactions at various [KaiA] and %ATP are fit to curves of the form FP (t) = $A\cos(2\pi T^{-1} t + \phi) + bt + c$ to extract the normalized amplitude (*100A/c*; dimensionless) of the oscillator; see Fig 2D for the periods (T) of the same reactions. Reactions with an amplitude $A < 0.5$ are considered to be non-oscillatory.

B Representative traces demonstrating the effect of %ATP at 1.25 μM KaiA; the polarization data are shifted vertically to avoid overlaps and horizontally to align the first peaks.

C Fluorescence polarization measurements of oscillatory reactions at three %ATP conditions in the presence of 0.0, 2.5, and 4.5 μM KaiC S431A/T431A (AA). The period (left) and amplitude (right) of the reactions are extracted using the same curve fit method as for panel A; non-oscillatory reactions are not shown.

D SDS–PAGE gel image of the supernatant from the KaiB-FLAG immunoprecipitation experiment.

E Simulated KaiC AA titration experiment using the modified Phong model. The points are model predictions, and the solid line is a linear fit. The dotted line is the linear fit to the experimental results (see Fig 3G). The simulations are carried out at 100% ATP, 1.5 μM KaiA, and $K_D = 10^{-3}$ μM condition.

F Comparison of the metabolic compensation property of the Phong model without (left) or with (right) a phosphorylation threshold at $K_D = 10^{-3}$ μM. The model exhibits phase decoherence at low %ATP without a phosphorylation threshold.

© 2020 The Authors

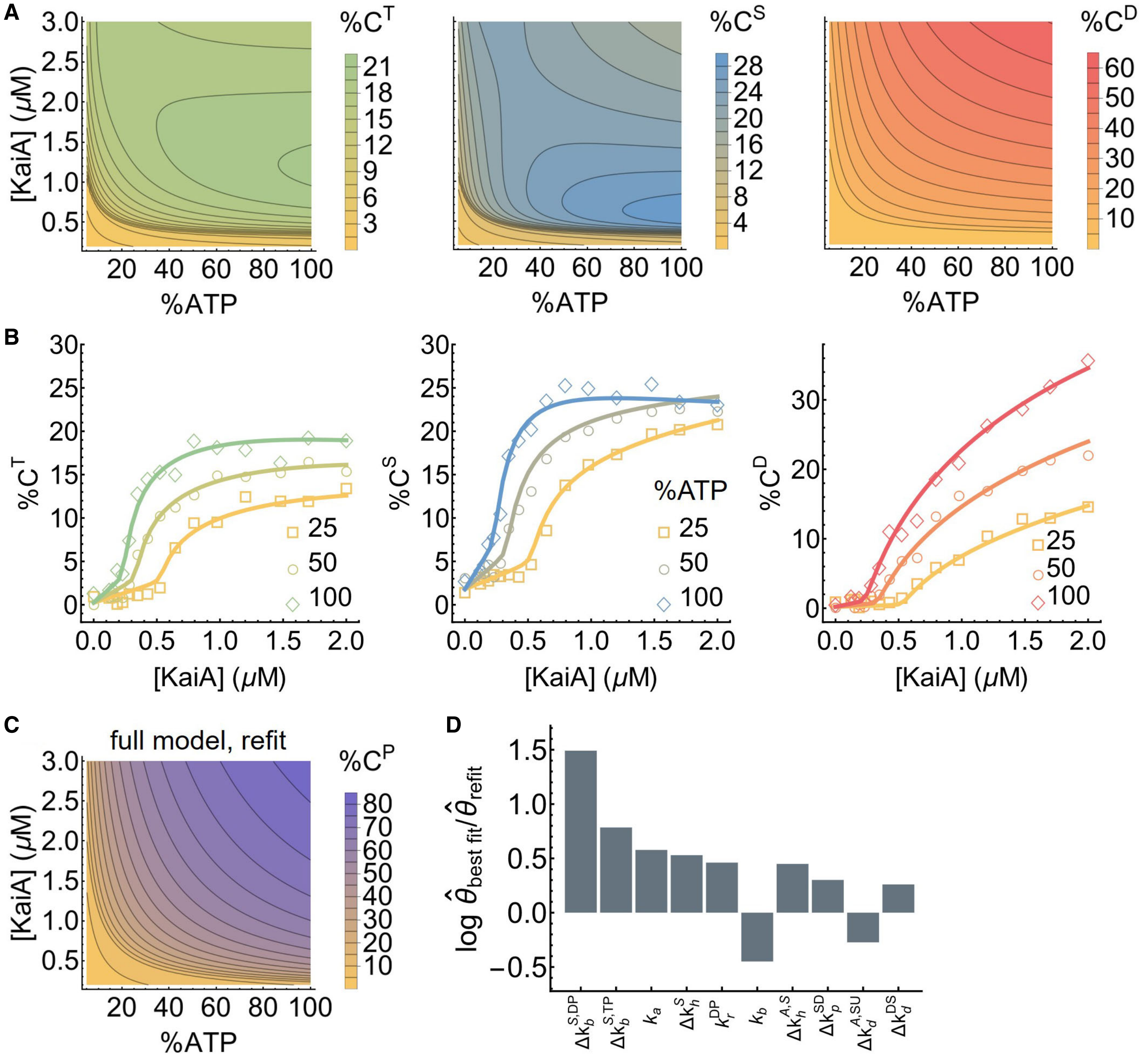

Figure EV4. KaiC stimulus–response relations.

A The steady-state stimulus–response relations for T, S, and D phosphoforms predicted by the model.

B The experimentally determined stimulus–response functions of the T, S, and D phosphoforms at three %ATP conditions; the curves are based on refitting the best fit to the steady-state measurements.

C The model-predicted stimulus–response relation of the total steady-state KaiC phosphorylation level as a function of %ATP and [KaiA] after refitting to the steady-state measurements.

D The differences in the log parameter values (base 10) of the best fit before and after refit. The differences are ordered by magnitude and only the 10 parameters (in the multiplicative-factor scheme) with the largest changes are shown.

 © 2020 The Authors

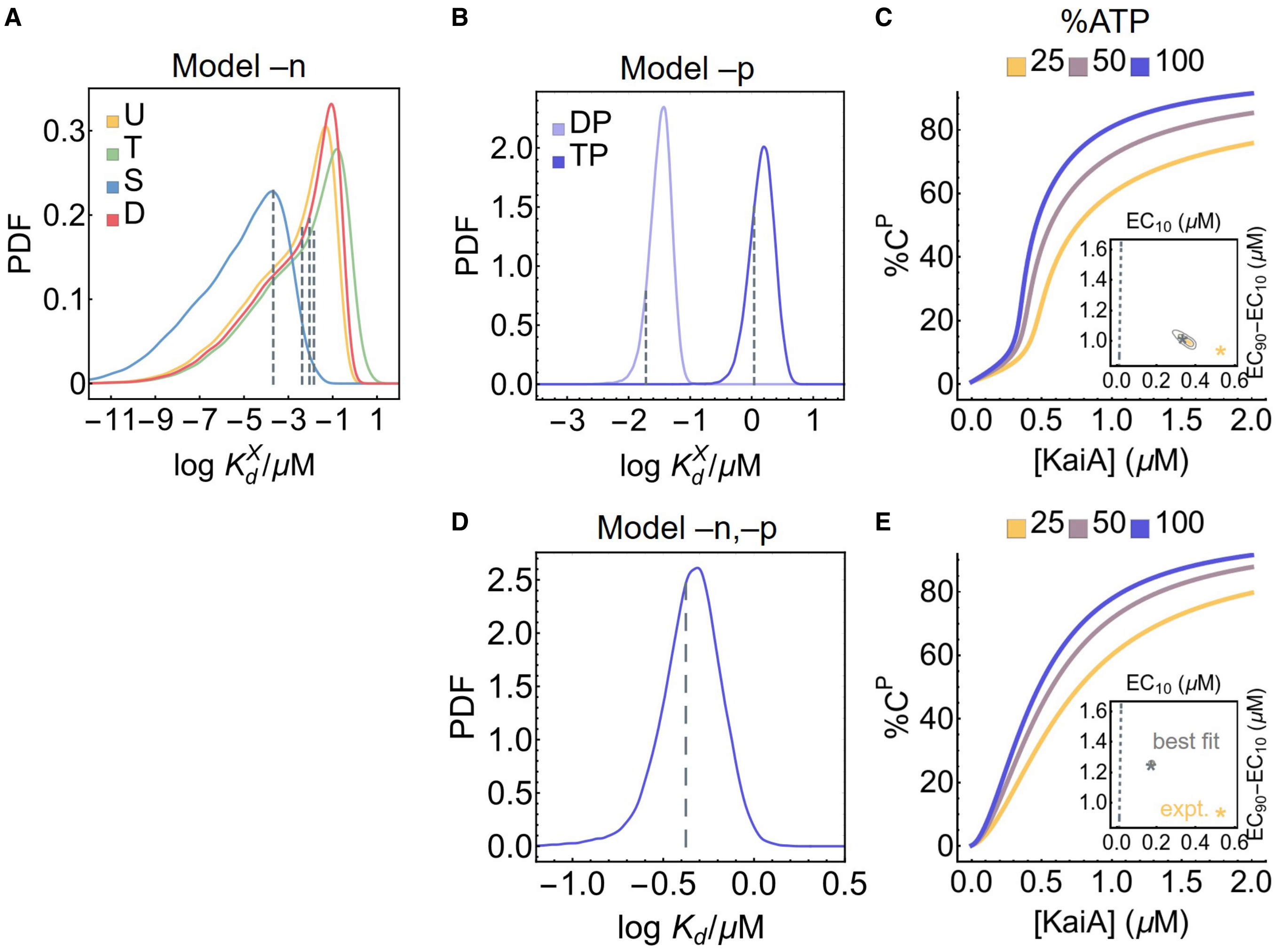

Figure EV5. KaiA binding affinities of simplified models.

A The posterior distributions for the KaiA dissociation constants as a function of KaiC phosphoform in model –n, where the KaiA on/off rates are decoupled from the nucleotide-bound states of KaiC. The dashed lines represent the best fit.

B Similar to (A), but for model –p, where the KaiA on/off rates are decoupled from the KaiC phosphoform; the dashed lines represent the best fit.

C Cross sections of the stimulus–response relation at three %ATP, computed using model –p. The inset represents the posterior distribution for the shape measures of the stimulus–response function at 25% ATP. The contours represent the 68% and 95% HDRs, and the gray star represents the model best fit. The shape of the stimulus–response function is quantified using two metrics: EC10, which quantifies threshold-like behavior, and EC90–EC10, which quantifies switch-like behavior. The shape measures of the experimentally determined stimulus–response function at 25% ATP is shown as the yellow star. The dashed line represents (EC10, EC90–EC10) =
(K/9, 80K/9), which characterizes the shape of a hyperbolic stimulus–response function [A]/(K + [A]) that has no switching or thresholding.

D Similar to (A), but for model –n,–p, where there is a single KaiA on/off rate in the model.

E Similar to (C), but for model –n,–p.

© 2020 The Authors
